# A global moderate resolution dataset of gross primary production of vegetation for 2000–2016

**DOI:** 10.1038/sdata.2017.165

**Published:** 2017-10-24

**Authors:** Yao Zhang, Xiangming Xiao, Xiaocui Wu, Sha Zhou, Geli Zhang, Yuanwei Qin, Jinwei Dong

**Affiliations:** 1 Center for Spatial Analysis, Department for Microbiology and Plant Biology, University of Oklahoma, Norman, OK 73019, USA; 2 Ministry of Education Key Laboratory for Biodiversity Science and Ecological Engineering, Institute of Biodiversity Science, Fudan University, Shanghai 200433, China; 3 State Key Laboratory of Hydroscience and Engineering, Department of Hydraulic Engineering, Tsinghua University, Beijing 100084, China; 4 Key Laboratory of Land Surface Pattern and Simulation, Institute of Geographic Sciences and Natural Resources Research, CAS, Beijing 100101, China

**Keywords:** Ecology, Carbon cycle, Climate change

## Abstract

Accurate estimation of the gross primary production (GPP) of terrestrial vegetation is vital for understanding the global carbon cycle and predicting future climate change. Multiple GPP products are currently available based on different methods, but their performances vary substantially when validated against GPP estimates from eddy covariance data. This paper provides a new GPP dataset at moderate spatial (500 m) and temporal (8-day) resolutions over the entire globe for 2000–2016. This GPP dataset is based on an improved light use efficiency theory and is driven by satellite data from MODIS and climate data from NCEP Reanalysis II. It also employs a state-of-the-art vegetation index (VI) gap-filling and smoothing algorithm and a separate treatment for C3/C4 photosynthesis pathways. All these improvements aim to solve several critical problems existing in current GPP products. With a satisfactory performance when validated against *in situ* GPP estimates, this dataset offers an alternative GPP estimate for regional to global carbon cycle studies.

## Background & Summary

Vegetation photosynthesis at the ecosystem scale, also known as the gross primary productivity (GPP), is the first step of CO_2_ entering the biosphere from the atmosphere. Over the past century, with the increasing carbon release from landcover change and fossil fuel burning, CO_2_ accumulation rate in land, ocean and atmosphere has continuously increased^1^. The increase of CO_2_ in Earth’s atmosphere is the major cause of global climate change^2^. Compared to the ocean sink, the global land sink exhibits much higher inter-annual variability and is also greatly affected by global climate change^1,3,4^. A major contribution of this high variability comes from GPP, as the photosynthesis process is vulnerable to droughts, heatwaves, floods, frost and other types of disturbances^5–8^. An accurate estimation of GPP will not only provide information about the ecosystem response to these extreme events, but also help to predict the future carbon cycle dynamics.

Multiple approaches to estimating GPP at a global scale have been developed over the past decades. Based on the fundamental theories used, they can be divided into four categories: the enzyme kinetic (process-based) models^9,10^, the light use efficiency (LUE) or production efficiency models^11,12^, machine learning techniques based on eddy covariance (EC) measurements^13,14^, and statistical models using solar-induced chlorophyll fluorescence (SIF)^15^. Amongst all these types of models, LUE models have been widely used because of its simple form and relatively long period of data availability. More importantly, it offers a balance between the temporal (usually 8-days) and spatial (usually up to 500 m or 250 m) resolution, which is suitable for regional to global scale studies. Most LUE models share a common form as follows^16^:

(1)GPP=LUE×APAR

where the APAR refers to the absorbed photosynthetically active radiation, and the LUE is an empirical factor that converts this absorbed energy to carbon fixed through photosynthesis process^17^.

The most widely used LUE GPP product is the MODIS GPP product^12^ (hereafter MOD17, GPP_MOD17_). However, site level validation studies have shown its moderate to low performance in capturing the seasonal and inter-annual variations^18^. Cross-site synthesis studies also reported large scale underestimates in GPP_MOD17_, especially for croplands^19,20^. Although improvements have been made in recent updates of MOD17 (C55 and C6), problems still exist, most related to the parameterization of the biome-specific lookup table and the climate drivers^21,22^. MOD17 assumes that the LUE remains constant for individual biome types, and the APAR is the energy absorbed by the entire canopy^12^. However, recent studies have shown that the photosynthetic capacity is not only affected by leaf quantity (amount of leaf area or leaf area index, LAI), but also by leaf quality (the photosynthetic rate of each individual leaf)^23,24^. The leaf quality is mostly related to the leaf chlorophyll content and the leaf nitrogen content^25,26^. There is not only a spatial variation of leaf quality within each biome due to the environment and nutrient availability^24^, but also a seasonal variation caused by the leaf’s phenological regulation^27^. MOD17 does not take leaf quality into consideration, and therefore exhibits a relatively low spatial and temporal representation of GPP when compared with *in situ* GPP estimations. Many recent studies suggest that the fraction of PAR absorbed by chlorophyll (fPAR_chl_) can better capture the seasonal variation of vegetation photosynthetic capacity and greatly improve the seasonal representation of GPP^28,29^. The vegetation photosynthesis model (VPM), developed based on this theory, showed superior performance with regards to site-level validations across a wide range of biome types^30–33^. A recent study also showed satisfactory performance (overall *R*^2^=0.82) of regional VPM GPP simulation in North America^34,35^ and the potential to apply this model to the entire globe.

**Figure 1:**
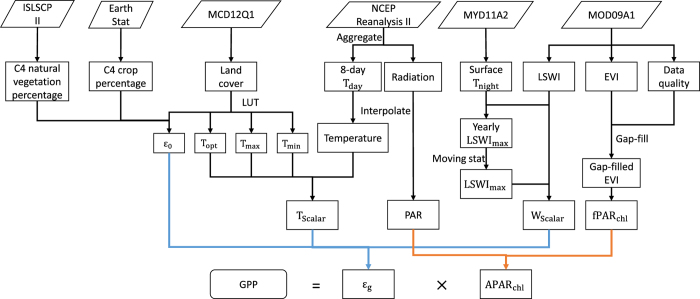
Datasets and workflow of VPM to calculate GPP. This figure is modified from ref. 34. ISLSCP II: International Satellite Land Surface Climatology Project, Initiative II; MODIS: Moderate Resolution Imaging Spectroradiometer; NCEP: National Centers for Environmental Prediction; EVI: enhanced vegetation index; LSWI: land surface water index; LST: land surface temperature; LUT: look-up table; T_day_: daytime air temperature; T_night_: nighttime land surface temperature; *ε*_0_: maximum light use efficiency; T_opt_: optimal temperature for photosynthesis; T_max_: maximum temperature for photosynthesis; T_min_: minimum temperature for photosynthesis; LSWI_max_: maximum LSWI during the growing season. T_scalar_: temperature limitation for photosynthesis; PAR: photosynthetically active radiation; W_scalar_: water limitation for photosynthesis; fPAR_chl_; fraction of PAR absorbed by chlorophyll; APAR_chl_: absorbed PAR by chlorophyll.

The objective of this study is to develop a new global GPP dataset that overcomes the limitation of MOD17. This dataset, dubbed VPM GPP V20, is based on an improved LUE theory which uses the energy absorbed by chlorophyll, and implemented to the entire globe during the MODIS era. The VPM algorithm utilized remotely sensed datasets together with reanalysis climate dataset and land cover classification. The schematic workflow of the VPM algorithm is shown in Fig. 1 and is described in detail in the Methods section. This dataset can serve as an alternative to MOD17 GPP product for regional and global carbon cycle studies.

## Methods

### Data processing

We used multiple datasets as the input of VPM. All datasets being used are summarized in Table 1, and detailed processing methods are described below.

### Vegetation indices and gap-filling

Enhanced Vegetation Index (EVI)^36^ and Land Surface Water Index (LSWI)^37^ are two major inputs for the VPM model, both of which are calculated from the MOD09A1 C6 500 m 8-day land surface reflectance dataset. To generate a continuous GPP product, there are two types of data gaps we need to consider: (1) missing observations: for some tiles at some DOY, e.g., year 2001, DOY 169, 177, because of the sensor calibration, no observations were acquired during those period; (2) bad-quality data: the data quality of this remote sensing reflectance measurement is often affected by the atmospheric conditions (e.g., cloud, aerosols), a rigorous quality check and gap-filling algorithm is required to generate a reliable time series of VI data for model simulation. Since EVI values are often lower if clouds or aerosols exist, while LSWI may be slightly higher, we used different strategies to gap-fill these two vegetation indices.

**Figure 2:**
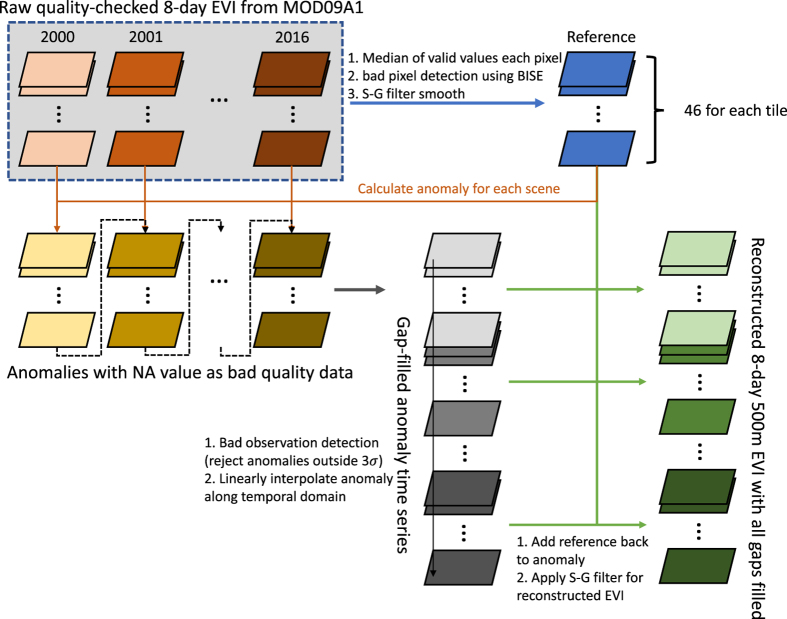
Flowchart showing the gap-filling and smoothing algorithm for reconstructing continuous EVI for 2000 to 2016. Different monochromatic colors represent different intermediate outputs, with different shades indicating different years.

The raw EVI calculated from the reflectance was first masked for all cloud, cloud shadow and aerosol affected regions based on the quality assurance (QA) layer in the MOD09A1 dataset (MOD35 cloud=‘clear’ (bit 0–1 equals ‘00’); aerosol quantity=‘low’ or ‘average’ (bit 6–7 equals ‘01’ or ‘10’))^8^ and then filled with an advanced gap-filling algorithm (Fig. 2). The median values of valid data for each day of year (DOY) across 17 years (2000–2016) were chosen as the reference for seasonal pattern. Under extreme conditions, some bad quality data may not be identified through the QA check, these data usually have a lower value and can be detected using the Best Index Slope Extraction algorithm^38^. After this process, gaps were filled with a linear interpolation and smoothed using a Savitzky-Golay filter. The reference will serve as a reliable baseline of the seasonal variation of EVI for each pixel. The novelty of this method is that, instead of linearly interpolate the EVI to fill the gaps within each year, we filled the gaps in seasonal anomalies (seasonal EVI−reference). This allows us to give a robust estimation when there is long period of missing/bad data during which EVI changes dramatically (Supplementary Information, Supplementary Fig. S1). To reconstruct the gap-filled EVI, we calculated the differences between EVI for one specific year and the corresponding DOY reference. The differences with missing values (generated by the quality check) were then gap filled and smoothed in the temporal domain. These gap-filled differences were added back to the reference to reconstruct the gap-filled and smoothed EVI. Using this method, we not only filled bad observations caused by atmospheric conditions, but also reconstructed the EVI for periods when no observations were made by the Terra satellite, e.g., in the year 2000 before DOY 49.

**Figure 3:**
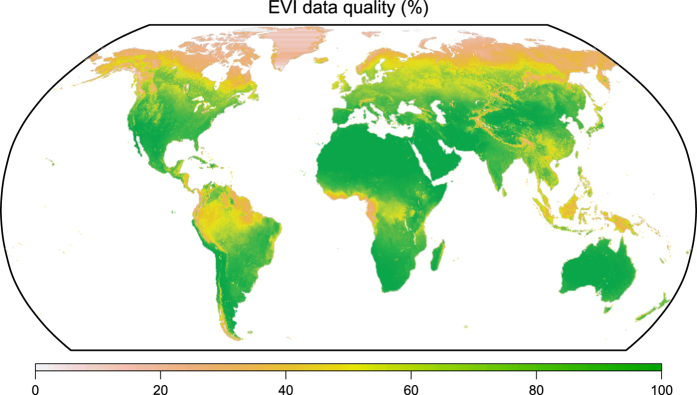
Average percentage of good EVI data (not gap-filled) for 2000–2016.

Figure 3 shows the average percentage of the good EVI data for the entire period. Gap-filled EVI data mostly locates in boreal regions, which is caused by high frequency of snow, clouds and aerosols; and tropical regions, which is caused by clouds and aerosols. No significant trend in data quality was found for most areas during 2002–2015 (Supplementary Information, Supplementary Fig. S2).

For LSWI which also suffers from missing observations in 2000, 2001 and 2016, we used the corresponding DOY scenes in 2001, 2002 and 2015 to fill the gaps in 2000, 2001 and 2016, respectively. This approach may introduce uncertainties due to the year to year variation of vegetation water condition and the biased estimation when cloud or snow exist. However, we believe it has limited impact on the GPP estimates since LSWI was not a factor for GPP but was used to indicate water stress. When cloud and snow exist, it usually corresponds to a higher LSWI value and the water stress is limited. We did not use the same algorithm for LSWI gap-filling because (1) LSWI is sensitive to surface water (snow, ice, flooding, etc.), and will exhibit a higher value which cannot be removed by using the BISE algorithm. (2) LSWI has higher variance which contains information of water stress and using this gap-filling algorithm may smooth this information. (3) atmospheric corruptions have limited effects on LSWI than EVI^8,30^. Future studies may consider using vegetation optical depth (VOD) as an indicator of water stress and upscale to higher spatial resolution^39^.

### C4 percentage for cropland and natural vegetation

C3/C4 plants have substantial differences in utilizing solar energies^40^. C4 plants do not have photorespiration and are less likely to have the light saturation effect^41^. Many previous studies have shown that C3/C4 plants should be treated differently in LUE models^42^. Herein lies one of the major causes for underestimation of GPP in cropland, since many models do not consider the C3/C4 difference.

Our previous VPM GPP product for North America did take C3 and C4 crop into consideration^34^. However, because of the data availability and spatial and temporal coverage, we did not used the Cropland Data Layer but made a simple assumption that the C3/C4 species are half-half for all cropland pixels. This is not accurate since, for example, the Mid-West corn belt grows much more corn (C4) than the Mississippi river basin. Since we aim to develop a global GPP product in this study, we use two static C4 vegetation maps (one for cropland, one for natural vegetation) for the entire globe. Although these maps are low in spatial resolution (Table 1) and lack inter-annual variation, they provide the best estimates of C4 vegetation distribution based on our current knowledge^43,44^. C4 crop and natural vegetation are considered separately. For C4 crop, we used the Earth Stat global major crop type distribution for the year 2000^44^. Among 19 major crop types, 4 are C4 crops (corn, millet, sorghum, sugarcane). This dataset provides the plant area percentage of each crop type for each 0.083 degree gridcell, through which we calculated the C4/C3 crop ratio:

(2)RatioC4=C4croppercentageTotalcroppercentage

where the Total crop percentage for the 0.083 gridcell was calculated from the MODIS landcover dataset (MCD12C1 C55). Within each 0.083 degree gridcell, all 500-m cropland pixels were considered to have a fixed C3/C4 crop ratio which allowed us to calculate the LUE using the area-weighted average of C3 and C4 LUE. However, this assumption introduces uncertainty when comparing simulated GPP with GPP estimates from cropland flux towers, since most sites are surrounded by either pure C3 or C4 crop. When averaged over a larger scale however, this mismatch may be negligible. We used the ISLSCP II C4 vegetation map for natural C4 vegetation distribution^43^ and assumed that all grassland, savannas, woody savannas and wetland within each 1°×1° gridcell share the same C3/C4 ratio. The corresponding LUE for these C3/C4 mixed pixels can be calculated based on their area-weighted averages.

### Climate data

For the global GPP simulation, we used the NCEP reanalysis II dataset. The daily downward shortwave radiation flux at surface (dswrf in Wm^−2^) was averaged over 8 days to match the model simulation interval. We used the average of daily maximum temperature (tmax.2m) and the daily mean temperature (air.2m) as daytime temperature and it was then averaged over each 8-day period. The aggregated datasets were in Gaussian grid (192×96) and further downscaled to the 500-m resolution using the same method that was described in previous publications^21,34^.

### Vegetation photosynthesis model (VPM) description

The VPM model follows the light use efficiency scheme and estimates GPP as the product of light absorption by chlorophyll of the vegetation (APAR_chl_) and the efficiency (*ε*_g_) that converts the absorbed energy to carbon fixed by plants through photosynthesis:

(3)GPP=APARchl×εg

where APAR_chl_ is calculated as a product of photosynthetically active radiation (PAR) and the fraction of PAR absorbed by chlorophyll (fPAR_chl_):

(4)APARchl=PAR×fPARchl

The fPAR_chl_ is calculated as a linear function of EVI, which is modified from previous model framework^32^:

(5)fPARchl=(EVI−0.1)×1.25

The coefficients 0.1 and 1.25 are used to adjust for sparsely vegetated or barren land and have been validated using the solar-induced chlorophyll fluorescence data (data not shown). *ε*_g_ in equation (3) is down-regulated by temperature limitation (T_scalar_) and water stress (W_scalar_) from its maximum value (*ε*_0_) which only differs by C3/C4 photosynthesis pathways:

(6)εg=ε0×Tscalar×Wscalar

Both T_scalar_ and W_scalar_ range from 0 to 1 and can be calculated as follows:

(7)Tscalar=(T−Tmax)×(T−Tmin)(T−Tmax)×(T−Tmin)−(T−Topt)2

(8)Wscalar=1+LSWI1+LSWImax

Where the T, T_max_, T_min_ and T_opt_ refer to the daytime mean temperature, maximum, minimum, and optimum temperature for photosynthesis, respectively. The last three parameters are biome-based and can be obtained from a look-up table (Table 2). The land cover product from MODIS (MOD12Q1) is used to provide biome information since this is the only annual land cover product with high spatial resolution and global coverage. The uncertainty of the land cover classification is not assessed but is supposed to have limited effect on the final GPP estimation since it only directly affects the temperature scalar and indirectly affects *ε*_0_. LSWI_max_ is the maximum LSWI during the snow-free period for each pixel each year. To eliminate potential bias, a temporal smoothing using nearby four years (two years before, two years after) is applied and calculates the second largest LSWI_max_ within this five-year period^34^.

### Code availability

The code for EVI gap-filling and VPM algorithms are available at https://github.com/zhangyaonju/Global_GPP_VPM_NCEP_C3C4.

## Data Records

The original VPM GPP V20 dataset is available at 500 m spatial resolution and 8-day temporal resolution. The entire earth land surface is divided into 290 subregions (tiles) under the sinusoidal projection. There are 46 GeoTiff files for each year each tile, each of which represents GPP for an 8-day average. The units are all in g C m^−2^ day^−1^ with a scalar factor of 0.001. Each file is also associated with a data quality layer: a Boolean value indicates whether the EVI is from the raw data (0) or gap-filled (1). We also provide two coarser spatial resolutions at 0.05°×0.05° and 0.5°×0.5° with a longitude-latitude projection under WGS84 datum. Three temporal resolutions, i.e., 8-day, monthly and annual, are available for 0.05°×0.05° and 0.5°×0.5° spatial resolution products. The units are g C m^−2^ day^−1^, g C m^−2^ month^−1^, and g C m^−2^ year^−1^, respectively. GPP products at 0.05°×0.05° and 0.5°×0.5° spatial resolutions can be accessed at (Data Citation 1); the raw 500 m 8-day GPP product together with the data quality layer can be accessed at (Data Citation 2). Because of the state-of-the-art gap-filling and smoothing algorithms applied, we can provide a continuous spatial temporal GPP estimate with no missing tiles.

**Figure 4:**
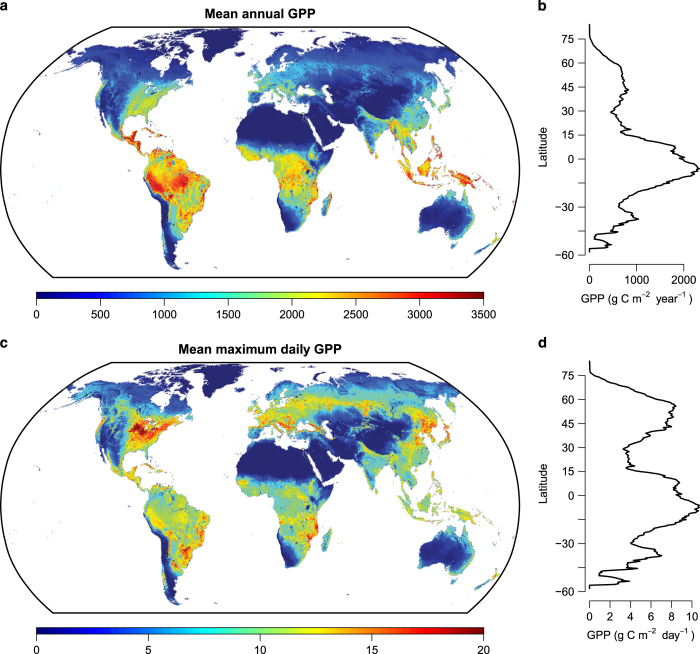
Spatial pattern of annual and maximum daily GPP for 2000–2016. (**a**) average annual GPP, (**c**) maximum daily GPP, latitudinal pattern of (**b**) average annual GPP, and (**d**) maximum daily GPP. Annual total GPP and maximum daily GPP within a year were averaged over the period of 2000 to 2016.

The global GPP estimation for the period 2000 to 2016 ranges from 121.60 to 129.42 Pg C year^−1^ with an increasing rate of ~0.39 Pg C year^−2^. The highest annual GPP occurs mostly in tropical regions, especially in the Amazon and Southeast Asia (Fig. 4a). This corresponds to the highest peak in annual GPP around the equator (Fig. 4b). The maximum daily GPP however, shows its highest value in the Midwest region of the United States. Other crop planting regions, e.g., central Europe, Northeast China, Southeast Africa and South America also show relatively high maximum daily GPP. High maximum productivity in these regions creates another peak around 50°N (Fig. 4d).

All other continents except South America and Oceania exhibit an increasing trend of annual GPP over the past 17 years (Table 3). The increasing rate is highest for Europe (0.89% year^−1^), followed by Asia (0.64% year^−1^), North America (0.61% year^−1^), and Africa (0.21% year^−1^). Annual GPP for Oceania did not show much change throughout the study period while South America experienced a significant decrease (−0.19% year^−1^).

## Technical Validation

### Comparison against eddy covariance flux tower

**Figure 5:**
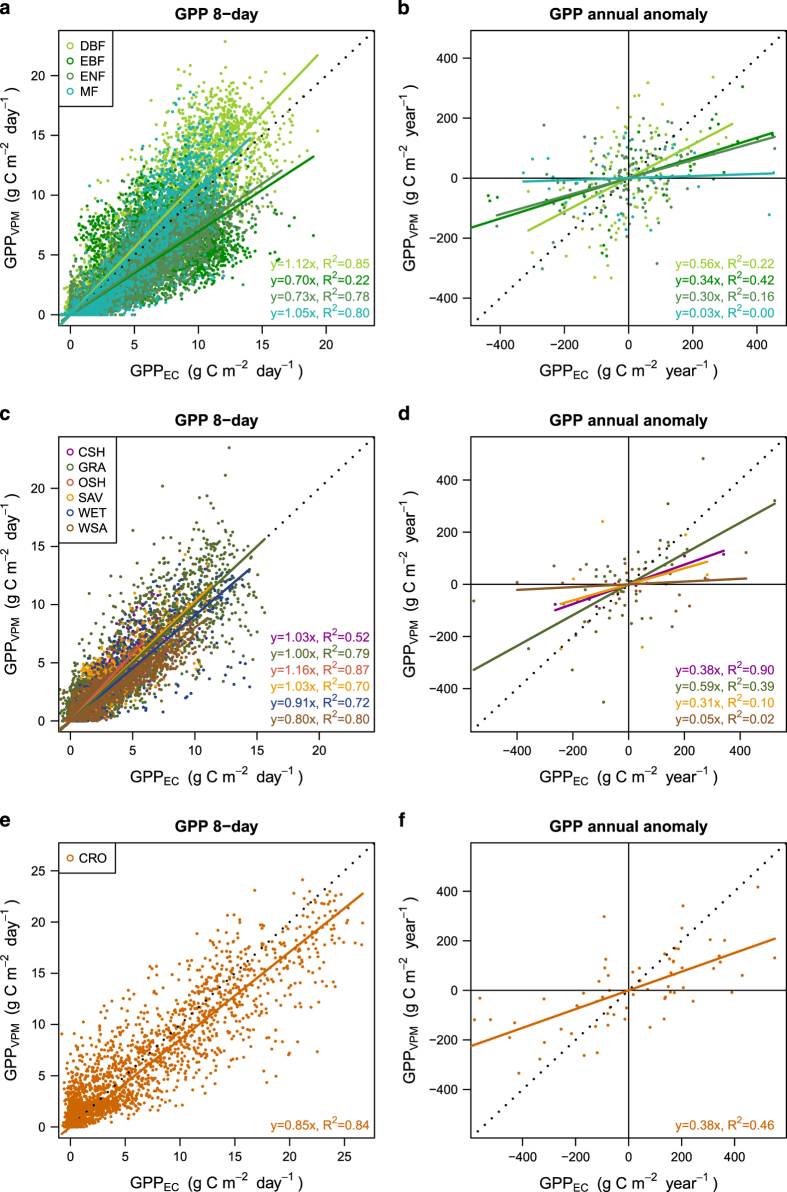
GPP validation against eddy covariance measurements for different biome types. Comparison of GPP for (**a**,**b**) forest, (**c**,**d**) non-forest and (**e**,**f**) cropland at 8-day scale across sites, and at annual scale in anomalies. Each point in (**a**,**c**,**e**) represents GPP estimates from VPM and EC for an 8-day period from one site; each point in (**b**,**d**,**f**) represents annual GPP estimates anomalies (GPP for one year minus GPP average over years for this site) for one year from one site; only sites with more than 5 years’ observations were used for annual anomaly comparison.

This GPP dataset was validated against 113 eddy covariance flux towers across the globe. These flux towers data were obtained from the FLUXNET 2015 Tier 1 dataset (2016 November release, http://fluxnet.fluxdata.org/data/fluxnet2015-dataset/), and only a small portion (23 out of 136) was excluded due to inconsistency between the flux tower footprint and MODIS pixels (Supplementary Information,Supplementary Table S1). GPP from the flux tower was calculated as an average from both daytime and nighttime partition methods after a rigorous data quality check. The validation was carried out at two scales: (1) at spatial and seasonal scale, we used all 8-day GPP estimates for all sites-years (*n*=28,378) and (2) at interannual scale, we calculated annual GPP anomalies for long-term sites from both EC estimates and VPM (more than 5 years of observation between 2000 and 2016, *n*=479) (Fig. 5).

The overall accuracy of the VPM GPP V20 dataset is relatively high with an *R*^2^ of 0.74 and a low RMSE of 2.08 g C m^−2^ day^−1^. A complete list of sites used and GPP comparisons is available in the Supplementary Information (Supplementary Table S1). For individual biome types, the VPM GPP product underestimated evergreen forest (~27% and 30% for ENF and EBF, respectively) and slightly underestimated cropland (~15%). For other biome types, the VPM GPP did not show a systematic bias for the spatial seasonal variation. Except ENF and CSH, most biome types showed relative high *R*^2^ values (>0.7) which means VPM GPP captures well the spatial and seasonal variations. In terms of interannual variation, VPM GPP did not show much agreement with EC-based GPP estimates. Across all biome types, VPM GPP showed less inter-annual variation compared with that from the EC tower. The *R*^2^ is low (<0.5) for most biome types. This low consistency in interannual variation may be caused by insensitivity of stress factors of VPM at an interannual scale, ecosystem memory effects which is not taken into consideration in VPM, and uncertainties in both GPP estimates.

### Comparison with other GPP datasets

**Figure 6:**
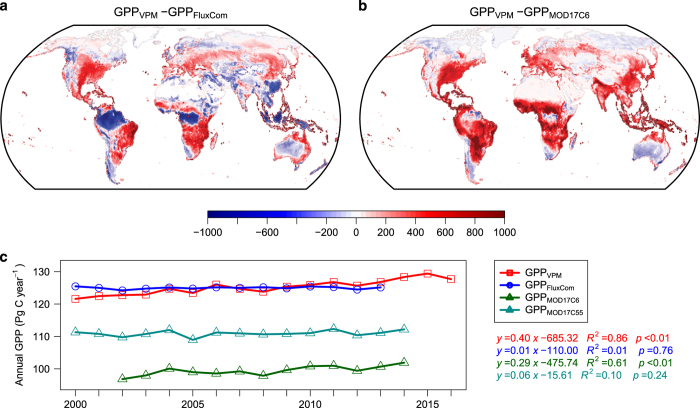
Comparison of GPP_VPM_ with other GPP products. Mean annual GPP difference between VPM and (**a**) FluxCom^14,61^ (for the period 2000–2013) and (**b**) MOD17A2H C6^62^ (MOD17C6 for the period 2002–2014) and (**c**) annual total GPP from VPM, FluxCom, MOD17 (both C6^62^ and C55 version^22^). Units for (**a**,**b**) are g C m^−2^ year^−1^. GPP_MOD17C6_ was not available for year 2000, 2001 and 2015 because of the missing tiles in the data distribution center. All comparisons were conducted at 0.5°×0.5° spatial resolution.

We also compared our GPP dataset with some other data-driven GPP products, e.g., FluxCom GPP, MOD17 (both C55 and C6). VPM showed higher annual GPP estimates in eastern and central US, mid-latitude Eurasia, and subtropical region in South America and Africa (Fig. 6a). These regions are mostly dominated by C4 cropland or grassland. VPM GPP is lower in tropical rainforest. When compared with MOD17C6, VPM gave higher estimations for most mid- to low-latitude regions, which is consistent with a recent study suggesting that MOD17C6 largely underestimates GPP for most ecosystems^20^.

The average global annual GPP estimates are similar in value for VPM and FluxCom, both around 125 Pg C year^−1^, though VPM showed a significant increasing trend (0.39 Pg C year^−2^) while FluxCom is almost stable during the past 14 years (Fig. 6c). Both MOD17 products showed lower GPP estimates, with a difference of ~14 Pg C year^−1^ for MOD17C55 and ~26 Pg C year^−1^ for MOD17C6, in comparison with VPM GPP. The C6 version of MOD17 also showed a significant increasing trend (0.29 Pg C year^−2^) that did not appear in the C55 version. This difference may be caused by using different climate data (GMAO/NASA (Global Modeling and Assimilation Office/National Aeronautics and Space Administration) and NCEP Reanalysis II) or the corrected sensor degradation effect in MODIS C6 data^45,46^.

**Figure 7:**
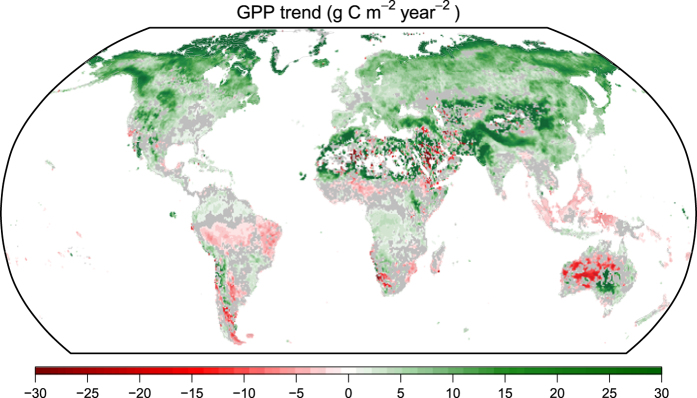
The GPP trend estimated from the seasonal Theil-Sen’s slope estimator^63,64^. Grey area indicates no significant trend at 0.05 level according to the seasonal Mann-Kendall test^65^. Very high and low values in Arabian Peninsula and central Australia may be caused by artifacts of the seasonal Theil-Sen’s slope method and is not evident in linear regression.

We also calculated the spatial pattern of GPP trend using the 0.5°×0.5° 8-day dataset as a further validation (Fig. 7). This spatial pattern is consistent with many previous studies: (1) high latitude regions and Qinghai Tibet Plateau experienced a continuous increasing GPP caused by warming and extended growing season^47–50^; (2) afforestation in Northern China^51,52^ and vegetation greening in southern Sahara Desert^53,54^ also lead to an increase in GPP; (3) Tropical forest in southeast Asia^55^ and southern part of Amazon^56^ where deforestation happened exhibited a decreasing trend of GPP.

Overall, GPP_VPM_ provides a reliable GPP estimation for non-forest natural vegetation and cropland by considering the C3/C4 vegetation ratio^57^. The underestimation for ENF and EBF may be related to the higher light use efficiency for diffused radiation^58^, but requires further testing. The total global GPP estimation is close to previous studies using different approaches^59,60^, and the trend of annual GPP increase are also in line with some other recent reports^4^.

## Usage Notes

The GPP for gridcells in coastal regions (in 0.05°×0.05 and 0.5°×0.5° spatial resolution products) are averaged over the entire gridcell but land only, therefore, the land area fraction is not needed when calculating the regional sum.

## Additional information

**How to cite this article:** Zhang, Y. *et al.* A global moderate resolution dataset of gross primary production of vegetation for 2000–2016. *Sci. Data* 4:170165 doi: 10.1038/sdata.2017.165 (2017).

**Publisher’s note:** Springer Nature remains neutral with regard to jurisdictional claims in published maps and institutional affiliations.

## Supplementary Material



Supplementary Information

## Figures and Tables

**Table 1:** Input datasets used to drive vegetation photosynthesis model (VPM) and their specifications.

**Data source**	**Dataset**	**Derived variables**	**Original spatial resolution**	**Original temporal resolution**	**Reference URL**
MODIS	MOD09A1 C6	EVI, LSWI	500 m	8-day	https://doi.org/10.5067/modis/mod09a1.006
	MYD11A2 C6	Nighttime LST (01:30 am overpass)	1 km	8-day	https://doi.org/10.5067/modis/myd11a2.006
	MCD12Q1 C51	Land cover type	500 m	Annual	https://lpdaac.usgs.gov/dataset_discovery/modis/modis_products_table/mcd12q1
NCEP-reanalysis II	Daily maximum/minimum air temperature	Daytime temperature	~1.875°×2°	daily	http://www.cpc.ncep.noaa.gov/products/wesley/reanalysis2/kana/reanl2-1.htm
	Radiation	Daily mean PAR	~1.875°×2°	daily	http://www.cpc.ncep.noaa.gov/products/wesley/reanalysis2/kana/reanl2-1.htm
Earth Stat	Major crop types distribution	C4 crop percentage	0.083°	invariant	http://www.earthstat.org/data-download/
ISLSCP II	C4 vegetation percentage map	C4 grassland percentage	1°×1°	invariant	https://doi.org/10.3334/ORNLDAAC/932
For acronyms please refer to captions in Fig. 1.					

**Table 2:** Biome specific lookup-table.

**IGBP class**	***ε***_**0**_ **(g C/mol APAR)**	**T**_**min**_ **(°C)**	**T**_**max**_ **(°C)**	**T**_**opt**_ **(°C)**
ENF	0.42	−1	40	20
EBF	0.42	−2	48	28
DNF	0.42	−1	40	20
DBF	0.42	−1	40	20
MF	0.42	−1	48	19
CSH	0.42	−1	48	25
OSH	0.42	1	48	31
WSA	0.42	−1	48	24
SAV	0.42 (C3) 0.63 (C4)	1	48	30
GRA	0.42 (C3) 0.63 (C4)	0	48	27
WET	0.42 (C3) 0.63 (C4)	−1	40	20
CRO	0.42 (C3) 0.63 (C4)	−1	48	30
URB	0.42	0	48	27
CNV	0.42 (C3) 0.63 (C4)	0	48	27
This table is adopted from ref. 34, where the *ε*_0_ values are from multiple site level studies^30,33,66,67^, and the temperature related parameters are adopted from Terrestrial Ecosystem Model^68,69^.				
ENF: evergreen needleleaf forest; EBF: evergreen broadleaf forest; DNF: deciduous needleleaf forest; DBF: deciduous broadleaf forests; MF: mixed forest; CSH: closed shrublands; OSH: open shrublands; WSA: woody savannas; SAV: savannas; GRA: grassland; WET: wetland; CRO: cropland; URB: Urban; NVM: cropland/natural vegetation mosaic.				

**Table 3:** Continental and global total GPP for each year.

**Year**	**Africa**	**Asia**	**Europe**	**North America**	**South America**	**Oceania**	**Global total**
2000	27.43	29.82	8.50	16.69	33.72	5.44	121.60
2001	27.69	30.52	8.64	17.22	33.43	4.96	122.46
2002	27.62	31.64	8.82	16.46	34.05	4.17	122.76
2003	27.57	31.18	8.54	17.33	34.02	4.29	122.93
2004	27.84	30.86	8.83	17.81	34.61	4.79	124.74
2005	26.93	31.00	8.90	17.88	34.27	4.43	123.41
2006	28.62	31.50	8.86	17.37	34.92	4.74	126.02
2007	28.20	31.76	9.04	18.03	33.28	4.39	124.70
2008	28.27	31.39	9.10	17.47	33.07	4.52	123.82
2009	28.80	31.82	9.07	17.23	33.92	4.54	125.38
2010	28.30	31.80	8.88	18.14	33.46	5.32	125.90
2011	28.54	32.21	9.43	17.26	33.52	5.82	126.79
2012	28.23	32.26	9.11	18.19	32.90	4.98	125.66
2013	29.09	33.00	9.48	17.89	32.89	4.48	126.81
2014	29.41	33.13	9.67	18.08	33.33	4.73	128.35
2015	28.02	34.42	9.79	19.05	33.73	4.42	129.42
2016	27.19	33.43	10.08	19.10	33.10	4.79	127.70
Units are in Pg C year^−1^.							
