# Author Correction: A global moderate resolution dataset of gross primary production of vegetation for 2000–2016

**DOI:** 10.1038/s41597-021-00854-6

**Published:** 2021-02-19

**Authors:** Yao Zhang, Xiangming Xiao, Xiaocui Wu, Sha Zhou, Geli Zhang, Yuanwei Qin, Jinwei Dong

**Affiliations:** 1 Center for Spatial Analysis, Department for Microbiology and Plant Biology, University of Oklahoma, Norman, OK 73019 USA; 2 Ministry of Education Key Laboratory for Biodiversity Science and Ecological Engineering, Institute of Biodiversity Science, Fudan University, Shanghai, 200433 China; 3 State Key Laboratory of Hydroscience and Engineering, Department of Hydraulic Engineering, Tsinghua University, Beijing, 100084 China; 4 Key Laboratory of Land Surface Pattern and Simulation, Institute of Geographic Sciences and Natural Resources Research, CAS, Beijing, 100101 China

Correction to: *Scientific Data* 10.1038/sdata.2017.165, published online 24 October 2017

In the Data Records section of this Data Descriptor, the data quality layer is incorrectly described as “raw data (0) or gap-filled (1)”. This should instead read “raw data (1) or gap-filled (0)”.

