# Supplementary material for: A global moderate resolution dataset of gross primary production of vegetation for 2000–2016
Source: Sci Data. 2017 Oct 24;4:170165. doi: 10.1038/sdata.2017.165 (PMC5667571; doi:10.1038/sdata.2017.165)
Supplement: Supplementary Information [file sdata2017165-s2.docx]

Supporting Information for “A global moderate resolution dataset of gross primary production of vegetation for 2000-2016”

Yao Zhang, Xiangming Xiao, Xiaocui Wu, Sha Zhou, Geli Zhang, Yuanwei Qin, Jinwei Dong

Figure S1. A schematic diagram showing our gap-filling method using one-year data of one pixel, and comparison with traditional linear interpolation method. When compared to the reference, the linear interpolation method did not correctly capture the seasonal dynamic of EVI compared to our gap-fill method. Please refer to the main text for detailed procedure of the algorithm.

Figure S2. Trend for EVI quality for 2002-2015 using the Theil-Sen’s slope estimator. Grey area shows no significant trend through Mann-Kendall test. Year 2000, 2001 and 2016 were not analyzed because of missing observations.

Table S1. 113 Fluxnet sites used for technical validation. IGBP: International Geosphere-Biosphere Programme land cover scheme, RMSE: root mean square error. rRMSE: relative root mean square error.

| Site ID | Site name | Latitude | Longitude | IGBP | Start year | End year | R^2^ | RMSE  (g C m^-2^ day^-1^) | rRMSE |
| --- | --- | --- | --- | --- | --- | --- | --- | --- | --- |
| AR-SLu | San Luis | -33.4648 | -66.4598 | MF | 2010 | 2010 | 0.79 | 0.67 | 0.09 |
| AT-Neu | Neustift | 47.1167 | 11.3175 | GRA | 2002 | 2012 | 0.76 | 2.12 | 0.38 |
| AU-Ade | Adelaide River | -13.0769 | 131.1178 | WSA | 2008 | 2008 | 0.92 | 0.42 | 0.11 |
| AU-ASM | Alice Springs | -22.283 | 133.249 | ENF | 2011 | 2013 | 0.81 | 0.29 | 0.41 |
| AU-Cpr | Calperum | -34.0021 | 140.5891 | SAV | 2011 | 2013 | 0.22 | 0.58 | 0.35 |
| AU-Cum | Cumberland Plains | -33.6133 | 150.7225 | EBF | 2013 | 2014 | 0.10 | 1.61 | 0.48 |
| AU-DaP | Daly River Savanna | -14.0633 | 131.3181 | GRA | 2008 | 2011 | 0.81 | 2.27 | 0.59 |
| AU-DaS | Daly River Cleared | -14.1593 | 131.3881 | SAV | 2008 | 2014 | 0.62 | 1.35 | 0.31 |
| AU-Dry | Dry River | -15.2588 | 132.3706 | SAV | 2012 | 2014 | 0.60 | 1.10 | 0.33 |
| AU-Emr | Emerald, Queensland, Australia | -23.8587 | 148.4746 | GRA | 2012 | 2013 | 0.42 | 1.19 | 0.97 |
| AU-Fog | Fogg Dam | -12.5452 | 131.3072 | WET | 2006 | 2007 | 0.48 | 1.19 | 0.46 |
| AU-Gin | Gingin | -31.3764 | 115.7138 | WSA | 2013 | 2014 | 0.49 | 0.84 | 0.27 |
| AU-GWW | Great Western Woodlands, Western Australia, Australia | -30.1913 | 120.6541 | SAV | 2013 | 2014 | 0.41 | 0.19 | 0.15 |
| AU-How | Howard Springs | -12.4943 | 131.1523 | WSA | 2003 | 2014 | 0.67 | 1.11 | 0.20 |
| AU-Rig | Riggs Creek | -36.6499 | 145.5759 | GRA | 2011 | 2014 | 0.52 | 1.12 | 0.37 |
| AU-Rob | Robson Creek, Queensland, Australia | -17.1175 | 145.6301 | EBF | 2014 | 2014 | 0.17 | 2.49 | 0.42 |
| AU-Stp | Sturt Plains | -17.1507 | 133.3502 | GRA | 2010 | 2014 | 0.70 | 0.94 | 0.71 |
| AU-TTE | Ti Tree East | -22.287 | 133.64 | OSH | 2013 | 2013 | 0.05 | 0.00 | 0.01 |
| AU-Tum | Tumbarumba | -35.6566 | 148.1517 | EBF | 2001 | 2014 | 0.58 | 1.30 | 0.15 |
| AU-Wac | Wallaby Creek | -37.4259 | 145.1878 | EBF | 2007 | 2008 | 0.24 | 2.14 | 0.38 |
| AU-Whr | Whroo | -36.6732 | 145.0294 | EBF | 2012 | 2014 | 0.52 | 0.78 | 0.23 |
| AU-Wom | Wombat | -37.4222 | 144.0944 | EBF | 2011 | 2012 | 0.71 | 1.22 | 0.19 |
| AU-Ync | Jaxa | -34.9893 | 146.2907 | GRA | 2013 | 2013 | 0.58 | 0.88 | 1.03 |
| BE-Bra | Brasschaat | 51.3092 | 4.5206 | MF | 2000 | 2014 | 0.82 | 1.75 | 0.44 |
| BE-Vie | Vielsalm | 50.3051 | 5.9981 | MF | 2000 | 2014 | 0.88 | 1.38 | 0.28 |
| BR-Sa3 | Santarem-Km83-Logged Forest | -3.018 | -54.9714 | EBF | 2001 | 2003 | 0.02 | 2.25 | 0.25 |
| CA-Man | Manitoba - Northern Old Black Spruce (former BOREAS Northern Study Area) | 55.8796 | -98.4808 | ENF | 2000 | 2003 | 0.92 | 0.54 | 0.30 |
| CA-NS1 | UCI-1850 burn site | 55.8792 | -98.4839 | ENF | 2003 | 2004 | 0.86 | 0.71 | 0.36 |
| CA-NS2 | UCI-1930 burn site | 55.9058 | -98.5247 | ENF | 2002 | 2004 | 0.86 | 0.69 | 0.39 |
| CA-NS3 | UCI-1964 burn site | 55.9117 | -98.3822 | ENF | 2002 | 2004 | 0.83 | 0.88 | 0.52 |
| CA-NS5 | UCI-1981 burn site | 55.8631 | -98.485 | ENF | 2002 | 2004 | 0.91 | 0.77 | 0.43 |
| CA-NS6 | UCI-1989 burn site | 55.9167 | -98.9644 | OSH | 2002 | 2004 | 0.91 | 0.72 | 0.64 |
| CA-NS7 | UCI-1998 burn site | 56.6358 | -99.9483 | OSH | 2003 | 2004 | 0.94 | 0.41 | 0.37 |
| CA-Qfo | Quebec - Eastern Boreal, Mature Black Spruce | 49.6925 | -74.3421 | ENF | 2004 | 2010 | 0.90 | 0.68 | 0.37 |
| CH-Dav | Davos- Seehorn forest | 46.8153 | 9.8559 | ENF | 2000 | 2014 | 0.69 | 1.67 | 0.54 |
| CH-Fru | Früebüel | 47.1158 | 8.5378 | GRA | 2007 | 2014 | 0.81 | 2.08 | 0.39 |
| CH-Lae | Laegeren | 47.4781 | 8.365 | MF | 2005 | 2014 | 0.88 | 1.96 | 0.39 |
| CN-Cha | Changbaishan | 42.4025 | 128.0958 | MF | 2003 | 2005 | 0.92 | 1.27 | 0.32 |
| CN-Dan | Dangxiong | 30.4978 | 91.0664 | GRA | 2004 | 2005 | 0.82 | 0.25 | 0.28 |
| CN-Din | Dinghushan | 23.1733 | 112.5361 | EBF | 2003 | 2005 | 0.26 | 1.73 | 0.43 |
| CN-Du2 | Duolun_grassland (D01) | 42.0467 | 116.2836 | GRA | 2007 | 2008 | 0.73 | 1.17 | 1.69 |
| CN-Ha2 | Haibei Shrubland | 37.6086 | 101.3269 | WET | 2003 | 2005 | 0.94 | 0.82 | 0.34 |
| CN-HaM | Haibei Alpine Tibet site | 37.37 | 101.18 | GRA | 2002 | 2004 | 0.91 | 0.59 | 0.35 |
| CN-Qia | Qianyanzhou | 26.7414 | 115.0581 | ENF | 2003 | 2005 | 0.77 | 1.18 | 0.25 |
| CN-Sw2 | Siziwang Grazed (SZWG) | 41.7902 | 111.8971 | GRA | 2011 | 2011 | 0.00 | 0.79 | 2.64 |
| CZ-BK1 | Bily Kriz forest | 49.5021 | 18.5369 | ENF | 2008 | 2008 | 0.84 | 1.17 | 0.21 |
| DE-Geb | Gebesee | 51.1001 | 10.9143 | CRO | 2001 | 2014 | 0.83 | 1.74 | 0.58 |
| DE-Gri | Grillenburg | 50.9495 | 13.5125 | GRA | 2004 | 2014 | 0.83 | 1.85 | 0.42 |
| DE-Hai | Hainich | 51.0792 | 10.453 | DBF | 2000 | 2012 | 0.90 | 1.93 | 0.43 |
| DE-Kli | Klingenberg | 50.8929 | 13.5225 | CRO | 2005 | 2014 | 0.82 | 1.81 | 0.54 |
| DE-Lkb | Lackenberg | 49.0996 | 13.3047 | ENF | 2011 | 2011 | 0.94 | 0.54 | 0.40 |
| DE-Obe | Oberb?renburg | 50.7836 | 13.7196 | ENF | 2008 | 2014 | 0.88 | 0.97 | 0.20 |
| DE-RuR | Rollesbroich | 50.6219 | 6.3041 | GRA | 2012 | 2014 | 0.85 | 1.77 | 0.38 |
| DE-Seh | Selhausen | 50.8706 | 6.4497 | CRO | 2008 | 2009 | 0.71 | 1.95 | 0.56 |
| DE-SfN | Schechenfilz Nord | 47.8064 | 11.3275 | WET | 2013 | 2014 | 0.86 | 0.98 | 0.42 |
| DE-Spw | Spreewald | 51.8923 | 14.0337 | WET | 2011 | 2014 | 0.94 | 1.14 | 0.25 |
| DE-Tha | Tharandt | 50.9636 | 13.5669 | ENF | 2000 | 2014 | 0.88 | 1.14 | 0.21 |
| DK-Sor | Soroe | 55.4859 | 11.6446 | DBF | 2000 | 2013 | 0.94 | 1.19 | 0.22 |
| ES-LgS | Laguna Seca | 37.0979 | -2.9658 | OSH | 2007 | 2008 | 0.80 | 0.49 | 0.41 |
| FI-Hyy | Hyytiala | 61.8475 | 24.295 | ENF | 2000 | 2014 | 0.92 | 0.87 | 0.28 |
| FI-Jok | Jokioinen | 60.8986 | 23.5135 | CRO | 2001 | 2002 | 0.90 | 1.22 | 0.92 |
| FR-Fon | Fontainebleau-Barbeau | 48.4764 | 2.7801 | DBF | 2005 | 2013 | 0.90 | 1.74 | 0.36 |
| FR-LBr | Le Bray (after 6/28/1998) | 44.7171 | -0.7693 | ENF | 2001 | 2008 | 0.63 | 1.82 | 0.41 |
| FR-Pue | Puechabon | 43.7414 | 3.5958 | EBF | 2001 | 2014 | 0.40 | 2.39 | 0.72 |
| GF-Guy | Guyaflux (French Guiana) | 5.2788 | -52.9249 | EBF | 2004 | 2014 | 0.02 | 1.78 | 0.18 |
| IT-BCi | Borgo Cioffi | 40.5238 | 14.9574 | CRO | 2005 | 2014 | 0.41 | 1.96 | 0.39 |
| IT-Col | Collelongo- Selva Piana | 41.8494 | 13.5881 | DBF | 2001 | 2014 | 0.91 | 2.00 | 0.52 |
| IT-Cp2 | Castelporziano 2 | 41.7043 | 12.3573 | EBF | 2013 | 2014 | 0.59 | 1.78 | 0.30 |
| IT-Cpz | Castelporziano | 41.7052 | 12.3761 | EBF | 2000 | 2008 | 0.57 | 2.28 | 0.42 |
| IT-Isp | Ispra ABC-IS | 45.8126 | 8.6336 | DBF | 2013 | 2014 | 0.93 | 0.95 | 0.18 |
| IT-La2 | Lavarone2 | 45.9542 | 11.2853 | ENF | 2001 | 2001 | 0.84 | 1.27 | 0.22 |
| IT-Lav | Lavarone | 45.9562 | 11.2813 | ENF | 2003 | 2014 | 0.75 | 1.66 | 0.26 |
| IT-MBo | Monte Bondone | 46.0147 | 11.0458 | GRA | 2003 | 2013 | 0.90 | 1.44 | 0.38 |
| IT-Noe | Arca di Noé - Le Prigionette | 40.6061 | 8.1515 | CSH | 2005 | 2013 | 0.39 | 1.08 | 0.34 |
| IT-PT1 | Parco Ticino forest | 45.2009 | 9.061 | DBF | 2002 | 2004 | 0.93 | 1.12 | 0.24 |
| IT-Ren | Renon | 46.5869 | 11.4337 | ENF | 2002 | 2013 | 0.83 | 1.17 | 0.29 |
| IT-Ro1 | Roccarespampani 1 | 42.4081 | 11.93 | DBF | 2001 | 2008 | 0.84 | 1.98 | 0.46 |
| IT-Ro2 | Roccarespampani 2 | 42.3903 | 11.9209 | DBF | 2002 | 2012 | 0.76 | 2.19 | 0.53 |
| IT-SR2 | San Rossore 2 | 43.732 | 10.291 | ENF | 2013 | 2014 | 0.81 | 0.74 | 0.12 |
| JP-MBF | Moshiri Birch Forest Site | 44.3869 | 142.3186 | DBF | 2005 | 2005 | 0.91 | 1.62 | 0.59 |
| JP-SMF | Seto Mixed Forest Site | 35.2617 | 137.0788 | MF | 2003 | 2006 | 0.77 | 1.68 | 0.39 |
| NL-Loo | Loobos | 52.1666 | 5.7436 | ENF | 2000 | 2013 | 0.91 | 0.73 | 0.16 |
| NO-Blv | Bayelva, Spitsbergen | 78.9216 | 11.8311 | SNO | 2008 | 2008 | 0.64 | 0.03 | 0.31 |
| RU-Fyo | Fyodorovskoye | 56.4615 | 32.9221 | ENF | 2000 | 2014 | 0.88 | 1.18 | 0.30 |
| SD-Dem | Demokeya | 13.2829 | 30.4783 | SAV | 2008 | 2009 | 0.83 | 0.36 | 0.27 |
| SN-Dhr | Dahra | 15.4028 | -15.4322 | SAV | 2012 | 2012 | 0.89 | 0.64 | 0.23 |
| US-AR1 | ARM USDA UNL OSU Woodward Switchgrass 1 | 36.4267 | -99.42 | GRA | 2010 | 2011 | 0.64 | 1.66 | 1.27 |
| US-AR2 | ARM USDA UNL OSU Woodward Switchgrass 2 | 36.6358 | -99.5975 | GRA | 2010 | 2011 | 0.64 | 1.14 | 1.16 |
| US-ARb | ARM Southern Great Plains burn site- Lamont | 35.5497 | -98.0402 | GRA | 2006 | 2006 | 0.92 | 0.89 | 0.46 |
| US-ARc | ARM Southern Great Plains control site- Lamont | 35.5465 | -98.04 | GRA | 2005 | 2006 | 0.94 | 1.10 | 0.37 |
| US-Blo | Blodgett Forest | 38.8953 | -120.6328 | ENF | 2000 | 2006 | 0.66 | 1.39 | 0.36 |
| US-Cop | Corral Pocket | 38.09 | -109.39 | GRA | 2002 | 2002 | 0.21 | 0.19 | 1.13 |
| US-Ha1 | Harvard Forest EMS Tower (HFR1) | 42.5378 | -72.1715 | DBF | 2000 | 2012 | 0.92 | 1.62 | 0.36 |
| US-KS2 | Kennedy Space Center (scrub oak) | 28.6086 | -80.6715 | CSH | 2004 | 2006 | 0.44 | 1.51 | 0.31 |
| US-Me2 | Metolius-intermediate aged ponderosa pine | 44.4523 | -121.5574 | ENF | 2002 | 2013 | 0.80 | 1.02 | 0.23 |
| US-Me6 | Metolius Young Pine Burn | 44.3233 | -121.6078 | ENF | 2011 | 2014 | 0.64 | 0.70 | 0.30 |
| US-MMS | Morgan Monroe State Forest | 39.3232 | -86.4131 | DBF | 2000 | 2014 | 0.91 | 1.88 | 0.41 |
| US-Ne1 | Mead - irrigated continuous maize site | 41.1651 | -96.4766 | CRO | 2002 | 2012 | 0.93 | 1.75 | 0.38 |
| US-Ne2 | Mead - irrigated maize-soybean rotation site | 41.1649 | -96.4701 | CRO | 2003 | 2012 | 0.93 | 1.65 | 0.41 |
| US-Ne3 | Mead - rainfed maize-soybean rotation site | 41.1797 | -96.4397 | CRO | 2002 | 2012 | 0.90 | 1.67 | 0.52 |
| US-NR1 | Niwot Ridge Forest (LTER NWT1) | 40.0329 | -105.5464 | ENF | 2000 | 2014 | 0.84 | 1.01 | 0.42 |
| US-ORv | Olentangy River Wetland Research Park | 40.0201 | -83.0183 | WET | 2011 | 2011 | 0.85 | 1.29 | 0.53 |
| US-Prr | Poker Flat Research Range Black Spruce Forest | 65.1237 | -147.4876 | ENF | 2011 | 2011 | 0.87 | 0.62 | 0.49 |
| US-SRG | Santa Rita Grassland | 31.7894 | -110.8277 | GRA | 2008 | 2014 | 0.75 | 0.84 | 0.73 |
| US-SRM | Santa Rita Mesquite | 31.8214 | -110.8661 | WSA | 2004 | 2014 | 0.79 | 0.46 | 0.54 |
| US-Syv | Sylvania Wilderness Area | 46.242 | -89.3477 | MF | 2002 | 2014 | 0.93 | 1.30 | 0.44 |
| US-Ton | Tonzi Ranch | 38.4316 | -120.966 | WSA | 2002 | 2014 | 0.57 | 0.96 | 0.39 |
| US-Tw1 | Twitchell Wetland West Pond | 38.1074 | -121.6469 | WET | 2013 | 2013 | 0.39 | 1.33 | 0.25 |
| US-Tw3 | Twitchell Alfalfa | 38.1159 | -121.6467 | CRO | 2014 | 2014 | 0.64 | 3.52 | 0.51 |
| US-Tw4 | Twitchell East End Wetland | 38.103 | -121.6414 | WET | 2014 | 2014 | 0.80 | 1.17 | 0.64 |
| US-Twt | Twitchell Island | 38.1087 | -121.653 | CRO | 2010 | 2014 | 0.78 | 2.01 | 0.56 |
| US-UMB | Univ. of Mich. Biological Station | 45.5598 | -84.7138 | DBF | 2000 | 2014 | 0.95 | 1.28 | 0.36 |
| US-UMd | UMBS Disturbance | 45.5625 | -84.6975 | DBF | 2008 | 2014 | 0.90 | 1.55 | 0.42 |

IGBP land cover abbreviations. ENF: evergreen needleleaf forest; EBF: evergreen broadleaf forest; DNF: deciduous needleleaf forest; DBF: deciduous broadleaf forest; MF: mixed forest; OSH: open shrubland; CSH: closed shrubland; GRA: grassland; WSA: woody savannas; SAV: savannas; WET: wetland; CRO: cropland; SNO: snow
